# The role of cortisol in immunosuppression in subarachnoid haemorrhage

**DOI:** 10.1186/s40001-023-01222-3

**Published:** 2023-08-29

**Authors:** Margaret E. Hoadley, James Galea, Navneet Singh, Sharon Hulme, David O. Ajao, Nancy Rothwell, Andrew King, Pippa Tyrrell, Stephen J. Hopkins

**Affiliations:** 1 Northern Care Alliance Research and Innovation, Salford Royal NHS Foundation Trust, Stott Lane, Salford, M6 8HD UK; 2 Ninewells Hospital and Medical School, University of Dundee, Ninewells, Dundee, DD1 9SY UK; 3 Division of Cardiovascular Sciences, Manchester Academic Health Sciences Centre, University of Manchester, Stott Lane, Salford, M13 9PT UK; 4 Atkinson Morley, Dept of Neurosurgery, St Georges Hospital, Blackshaw Rd, London, Sw17 0QT UK; 5 Stockport NHS Foundation Trust, Stepping Hill Hospital, Poplar Grove, Hazel Grove, Stockport, SK2 7JE UK; 6 Faculty of Life Sciences, University of Manchester, AV Hill Building, Oxford Road, Manchester, M13 9PL UK

**Keywords:** Cytokines, Immune response, Immunosuppression, Cortisol, RU-486, Infection

## Abstract

**Background:**

We sought to determine the extent to which cortisol suppressed innate and T cell-mediated cytokine production and whether it could be involved in reducing peripheral cytokine production following subarachnoid haemorrhage (SAH).

**Methods:**

Whole blood from healthy controls, patients with SAH and healthy volunteers was stimulated with lipopolysaccharide (LPS), to stimulate innate immunity, or phytohaemagglutinin (PHA), to stimulate T cell-mediated immunity. Varying concentrations of cortisol were included, with or without the cortisol antagonist RU486. Concentration of interleukin-6 (IL-6), IL-1β and tumour necrosis factor-alpha) TNFα were determined as a measure of innate immunity. IL-6, IL-17 (interferon gamma) IFNƔ and IL-17 were determined as an indicator of T cell-mediated immunity.

**Results:**

Suppression of innate responses to LPS was apparent in whole blood from SAH patients, relative to healthy controls, and TNFα production was inversely correlated with plasma cortisol concentration. Cytokine production in whole blood from healthy volunteers was inhibited by cortisol concentrations from 0.33 µM, or 1 µM and above, and these responses were effectively reversed by the cortisol antagonist RU-486. In SAH patients, RU-486 reversed suppression of innate TNF-α and IL-6 responses, but not IL-1ß or T cell-mediated responses.

**Conclusion:**

These data suggest that cortisol may play a role in reducing innate, but not T cell-mediated immune responses in patients with injuries such as SAH and that cortisol antagonists could be effective in boosting early innate responses.

## Background

An increased incidence of infection has been reported after acute central nervous system injuries such as stroke [6, 14, 46], traumatic brain injury [21, 26, 50], spinal cord injuries [32, 40] or subarrachnoid haemorrhage (SAH; [18]). Infections increase mortality and morbidity [31] and generally worsen clinical outcomes in brain-injured patients [22, 24]. It has been shown that there is a 28% incidence of sepsis in SAH patients and septic patients have a mortality of 52.5% compared to 16% in non-septic patients [20]. Although exposure to infectious agents is an important determinant, reduced host immune responsiveness is potentially an important factor.

Suppression of peripheral immune responses may be critical in predisposing patients to infections following stroke ([9, 12] or SAH [36]. Several mechanisms have been proposed to account for this apparent reduction in immune responsiveness, including sympathetic and parasympathetic neural regulation [2, 49] and activation of the hypothalamic–pituitary–adrenal (HPA) axis [51, 52]. We have reported that production of cytokines by peripheral blood leukocytes, in response to bacterial lipopolysaccharide (LPS), was reduced in individuals that had suffered a stroke [16]. Since blood leukocytes are not subject to direct neural innervation, the close correlation between suppression and plasma cortisol implicates hormonal involvement [15, 16]. Furthermore, in a trial of interleukin-1 receptor antagonist (IL-1Ra), designed to investigate post-stroke inflammation, we observed that plasma cortisol concentrations and immunosuppression were both reversed by IL-1Ra [38].

Since immune suppression had been noted in a pilot study of SAH patients [36], we have used ongoing studies in SAH to confirm these observations, by challenging the capacity of innate and adaptive immune cells to respond. We further examined whether there is a relationship between immune suppression and plasma cortisol in these patients, as seen in stroke. We then examined whether pathophysiologically relevant concentrations of cortisol could suppress both innate and T cell mediated cytokine induction, and the potential for antagonising suppression using the glucocorticoid receptor (GR) antagonist, RU-486, which has been shown to cross the blood brain barrier in studies into epilepsy and brain cancer [8, 53]. Subsequently we tested if RU-486 could restore innate and T cell mediated cytokine induction in blood from patients that had suffered a SAH.

## Materials and methods

### Blood collection from patients and controls

Patients were recruited from one of two studies at Salford Royal Foundation Trust to address the research objectives [23, 37]. The numbers of patients in the study were limited to those that we were able to recruit from these ongoing studies. The study was therefore essentially exploratory but, to increase the power to identify differences we obtained twice the number of controls, compared to numbers of patients.

#### Immune suppression following SAH

To assess the degree of immune suppression following SAH, 5 mL blood samples were collected in a tube containing 50 µL of heparin at 1000 IU/mL (Heparin Sodium 1000 IU/mL, Wockhardt UK Ltd, Wrexham, UK) from 11 patients up to 72 h after SAH and from 22 age- and sex-matched healthy controls. Patient samples were collected between 7:18 h and 19:22 h with a median collection time of 11:30 am. Control samples were collected between 8:10 h and 16:30 h with a median collection time of 10:30 am. The study was approved by the Salford and Trafford Local Research Ethics Committee and the Research Ethics Committee in Cardiff, Wales [23].

#### Impact of IL-1Ra and glucocorticoid inhibition on immune suppression

To evaluate the impact of RU-486 and IL-1Ra on immune suppression, participants were recruited from patients with SAH participating in a randomised, open-label, single-blinded, phase II study of sub-cutaneous IL-1Ra (EudraCT: 2011-001855-35; [37]). Blood was collected on admission (day 0), from 45 patients that were either untreated or just before the start of administration of IL-1Ra (100 mg administered subcutaneously, twice daily in 19 of these patients) and again at day 3. Ethical approval was obtained from the National Research Ethics Committee (11/NW/0390) and the Medicine and Healthcare products Regulatory Agency (MHRA).

For both studies, patients ≥ 18 years were eligible if they had radiologically confirmed aneurysmal SAH, there was no evidence of concurrent infection and consent could be obtained within 72 h of ictus. In all studies the tubes of blood were inverted 5–8 times to mix and kept at room temperature for a standard period of 1 h before processing.

### Blood collection from healthy volunteers

Venous blood was collected from 14 healthy human volunteers (8 females and 6 males), with a mean age of 44.8 years (± 14), who agreed to participate in the study and reported no symptoms of infection at the time of blood collection. A 30 mL sample was added to tubes containing 3000 IU pyrogen-free heparin. Informed consent was obtained from each subject, and the study was approved by the National Research Ethics Service Committee, North West.

### Preparation of cortisol and RU-486

A 1 mg/mL solution of cortisol, as hydrocortisone (H0888, Sigma, Poole, UK) was prepared in 100% ethanol and diluted in Roswell Park Memorial Institute medium (RPMI: Life Technologies, Paisley, UK) to 100 µM (3.6% ethanol) and stored at − 70 °C. For experimental use an aliquot was diluted in RPMI to give a range of cortisol concentrations from 0.01 to 1 µM, with resultant ethanol concentrations ranging from 0.0036 to 0.036%. Initial experiments demonstrated that concentrations of alcohol below 1% do not significantly affect cytokine production.

RU-486, as mifepristone (M8046, Sigma, Poole, UK), was diluted in 100% ethanol to 1 mM, and further diluted to 0.2 mM in RPMI (20% ethanol) for storage at − 70 °C. For experimental use, this was further diluted to at least 10 µM. The maximum concentration of ethanol in blood samples incubated with cortisol and RU-486 was 1.04%.

### Incubation of whole blood, from patients and their healthy controls, with LPS

To activate responses from cells of the innate immune system, whole blood volumes of 1.25 mL (from SAH patients or controls), were mixed with 1.25 mL RPMI, to which LPS had been added to a final concentration of 100 ng/mL. 1 mL volumes were added in duplicate to wells of 12-well multi-well plates (150628, SLS Ltd, Wilford, UK). Control wells, with blood plus RPMI only, were included and the dishes incubated for 24 h at 37 °C, 5% CO_2_. After 24 h the contents of each well were transferred to 1.5 mL microcentrifuge tubes (StarLab Group, Milton Keynes, UK) and centrifuged at 13400*g* for 10 min. The supernatants were separated and stored at − 70 °C until analysis.

### Incubation of whole blood, from healthy volunteers, with LPS or PHA-L and cortisol

In addition to activating cells of the immune system, as described above, whole blood volumes of 1.25 mL were mixed with 1.25 mL RPMI, to which either PHA-L (L2769, Sigma, Poole, UK) was added to a final concentration of 5 µg/mL, to induce activation of T cells. Cortisol was added to final concentrations of 0.01 to 1 µM. Control tubes of blood plus RPMI only, or RPMI with 100 ng/mL LPS or 5 µg/mL PHA-L only, were also included. The different combinations were added in duplicate 1-mL volumes, to wells of 12-well multi-well plates and incubated as above. After 24 h, the contents of each well were centrifuged and stored as described above.

### Incubation of whole blood from healthy volunteers with LPS or PHA-L, cortisol and RU-486

Equal volumes of blood and RPMI, to which PHA-L or LPS had been added, were prepared as described above. Cortisol was added to a final concentration of 1 µM, ± RU-486 to a final concentration of 1, 3.3 or 10 µM. Tubes with blood plus RPMI, or LPS and PHA-L only, were prepared as controls. The different combinations were added in duplicate 1 mL volumes, to wells of 12-well multi-well plates, before incubation, centrifugation and storage as described above.

### Incubation of whole blood from patients in the IL-1Ra trial with LPS or PHA-L and RU-486

Equal volumes of blood and RPMI, to which LPS or PHA-L had been added, were prepared as described above. Two tubes were prepared for LPS and two for PHA-L. To one LPS tube and one PHA tube, RU-486 was added at a concentration of 10 µM. Control tubes with blood plus RPMI only, and RPMI plus RU-486 were also included. All treatments were incubated for 24 h at 37 °C, 5% CO_2,_ before centrifugation and storage as described above. Blood was taken from patients up to 72 h after haemorrhage, before administration of IL-1Ra or placebo (day 0) and at day 3, after at least 24 h administration of IL-1Ra or placebo.

### Cytokine and cortisol measurement

Supernatants were thawed rapidly at 37 °C before measurement of cytokines. Where blood had been activated with LPS, to stimulate innate immunity, concentrations of IL-6, IL-1β, and TNF-α were measured, using Bio-Plex^®^-based multiplex assays (Bio-Rad Hemel Hempstead, UK), since these cytokines are produced by cells involved in innate immune responses to LPS. Where blood had been activated with PHA-L, to stimulate T cell-mediated immunity, concentrations of IL-6, IL-4, IFN-γ and IL-17 were measured using enzyme-linked immunosorbent assays (ELISA) or multiplex assays, since these cytokines are produced by T cells in response to PHA. IL-6 and IFN-γ ELISAs were used for supernatants from PHA-L-stimulated blood from healthy volunteers only: the multiplex system was used for supernatants from patient studies. IL-4 ELISAs were used for PHA-L-stimulated supernatants from both healthy volunteers and patients in the IL-1Ra trial. For both ELISAs and multiplex assays, laboratory standards were standardised against international standards provided by NIBSC, South Mimms, UK (IL-6 code:89/548, IL-4 code 88/656, IFNƳ code 87/586, IL-17 code 01/420, TNFα code 12/154, IL-1β code 86/680).

For multiplex assays, analytes were captured on carboxyl-coated polystyrene beads or Bio-Plex Pro Magnetic COOH beads (Bio-Rad, Hemel Hempstead, UK) that had been coupled to specific monoclonal antibodies, using the Bio-Plex amine coupling kit (171-406001, Bio-Rad). Capture antibodies were the coating monoclonal antibodies from the following reagent sets: anti-IL-6 (Pelikine, M191604), anti-IL-1β (Pelikine, M193404), anti-TNF-α (Pelikine, M192304) and anti-IFN-γ (Pelikine Compact kit, M1933) (all from MAST Diagnostics, Bootle, UK), and anti-IL-17 Duo set (DY317, R&D Systems, Abingdon UK). The analytes were secondarily bound with specific monoclonal antibodies conjugated with biotin from corresponding reagent sets, prior to addition of streptavidin, conjugated with phycoerythrin (016-110-084; Stratech Scientific Ltd., Newmarket, UK). Assays were carried out in Millipore-Multiscreen HTS- 96-well filter plates (Millipore, Watford, UK) or Bio-Plex Pro flat bottomed plates (Bio-Rad, Hemel Hempstead, UK). Results were read on a Bio-Plex 200 suspension array system and analysed using Bio-Plex Manager Software V6.1 (Bio-Rad).

ELISAs were carried out using Pelikine Compact Cytokine ELISA kits (Mast Diagnostics, Bootle, UK) for IL-6 (CLB-M1916), IL-4 (CLB-M1914) and IFN-γ (CLB-M19133), were read on a Wallac Victor 1420 Plate Reader (Perkin Elmer, Cambridge UK) and analysed using Delta Soft Microplate analysis software (Biometallics Inc. Princeton, USA). Quality controls, containing known concentrations of analytes, were included in each assay to determine inter-assay variability. Plasma cortisol was measured by the routine clinical biochemistry service, using an electrochemiluminescent competitive immunoassay on a Roche Modular E170 analyzer.

### Statistical analysis

For analysis of data from healthy volunteers (Figs. 2 and 3) repeated measures analysis of variance (ANOVA) was used to compare each treatment group with the positive (e.g. blood sample with LPS or PHA-L only) or negative (blood sample + RPMI only) control group. This was followed by Dunnett’s post hoc test to account for multiple comparisons with the control group. Differences with a confidence level of 95% were considered to be statistically significant (*p* < 0.05). The relative inhibition of cytokine production by cortisol, as well as reversal of inhibition with RU-486 was calculated and represented as medians. Bonferroni’s post hoc test was used for the 24 vs. 48 h multiple comparisons tests (see Fig. 2D–F), with alpha set at 5.0%. For the analysis of data from SAH patients and their matched controls, Wilcoxon matched-pairs signed rank test was used for comparisons within groups. For comparisons between groups (patients vs. controls or placebo vs. IL-1Ra), a Mann–Whitney test was used. Spearman rank correlation analyses were applied for univariate associations. All statistical analyses were carried out using GraphPad Prism software (GraphPad Software, Inc., La Jolla, CA, USA).

## Results

### Innate immune cytokine induction is suppressed in patients with SAH

**Fig. 1:**
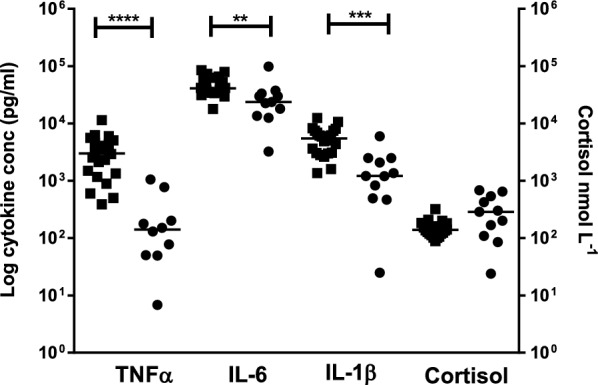
The effect of SAH on LPS-induced cytokine production and plasma cortisol concentration. Cytokine concentrations were measured in blood supernatants from controls and patients, following incubation with LPS. Cortisol at the time of blood collection was also measured in plasma from these controls and patients. Median values are indicated by the horizontal bars. Statistical significance is indicated at *p* < 0.01 (**), *p* < 0.001 (***) and *p* < 0.0001 (****) (Mann–Whitney)

In response to LPS there was significantly reduced production of TNF-α (*p* < 0.0001, 94%), IL-6 (*p* < 0.01, 39.1%) and IL-1β (*p* < 0.001, 78.1%) in blood from patients with SAH compared to age- and sex-matched controls (Fig. 1). The median plasma cortisol concentration in patients (288 nmol/L) was not significantly greater than in controls (139 nmol/L) but the distribution was significantly wider and 5 of 11 (45%) patients had cortisol levels higher than the patient group median, compared to 1 of 22 (4.5%) controls (Fig. 1). Plasma cortisol concentration was significantly inversely correlated with TNF-α (*r* = − 0.69, *p* < 0.05) in supernatants from LPS-stimulated blood cultures, although correlations with IL-1ß (*r* = 0.28), IL-6 (*r* = − 0.58), IL-8 (*r* = − 0.34) and IL-10 (*r* = − 0.51) were not statistically significant**.**

### Cortisol suppresses innate and T-cell cytokine induction in blood samples from healthy volunteers

**Fig. 2:**
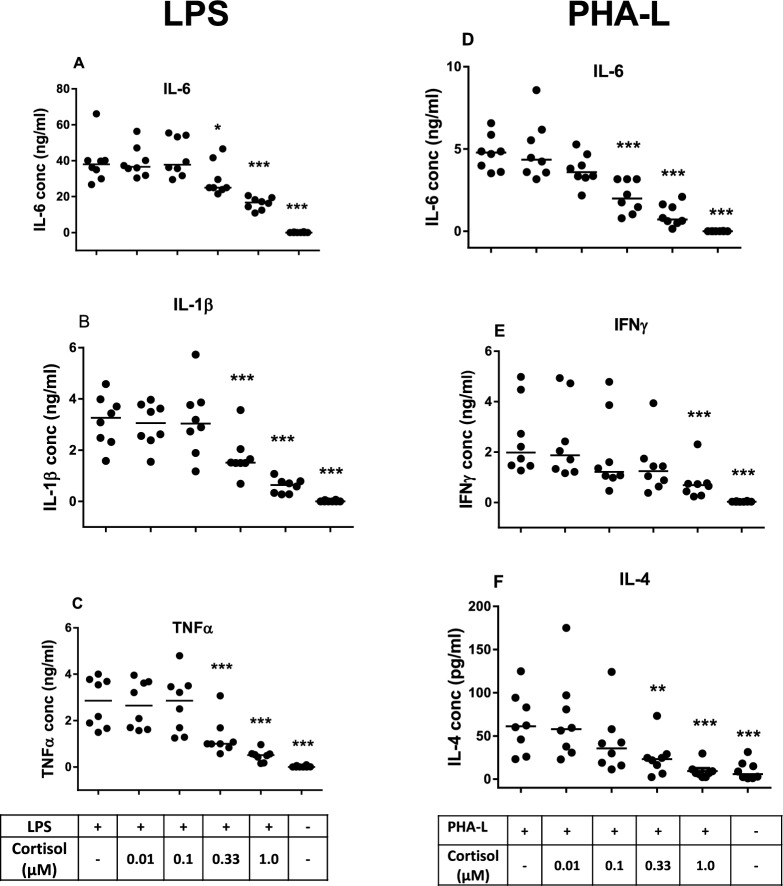
Cortisol suppresses LPS- and PHA-L-induced production of cytokines in healthy human blood. Cortisol was added at increasing concentrations to blood stimulated with LPS (**A**–**C**) or PHA (**D**–**F**) for 24 h. Median values are indicated by the horizontal bars. Statistical significance is indicated at *p* < 0.05 (*), *p* < 0.01 (**), *p* < 0.001 (***) and *p* < 0.0001 (****), relative to LPS or PHA-L-stimulated positive controls (repeated measures ANOVA and Dunnett’s multiple comparison test)

Cytokine production varied between individual healthy volunteers, but when cortisol was added to blood cultures, in the presence of LPS or PHA-L, it had a significant inhibitory effect for each of the cytokines measured (Fig. 2). This was apparent from concentrations of ≥ 0.33 μM cortisol for all measured cytokines except IFN-γ, which was inhibited at 1 µM cortisol.

### RU-486 reverses the suppressive effects of cortisol on innate and T cell-induced cytokine production in blood samples from healthy volunteers

**Fig. 3:**
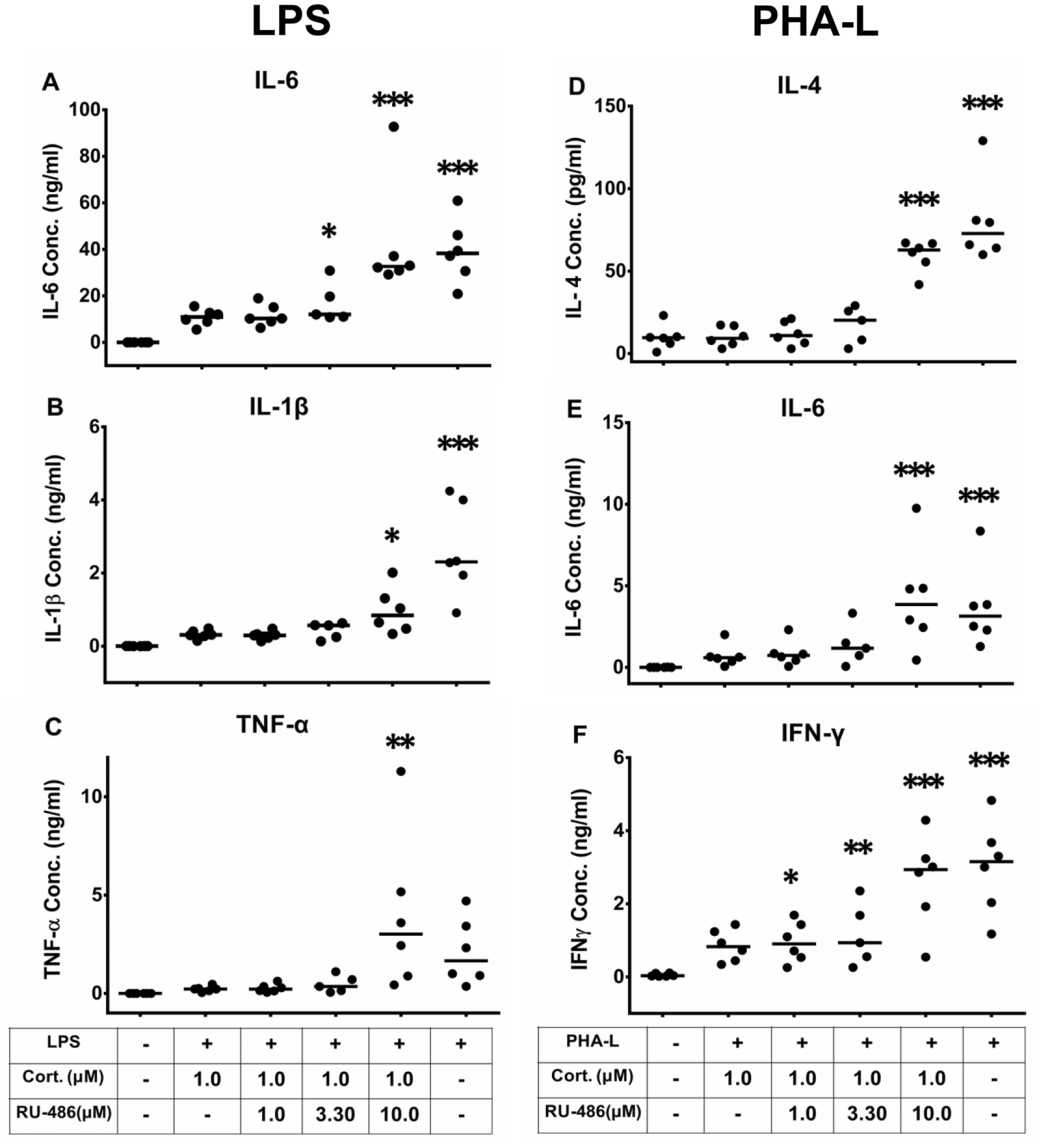
RU-486 reverses cortisol suppression of cytokine production in both LPS- and PHA-L-stimulated human blood. Production of IL-6 (**A**), IL-1β (**B**) and TNF-α (**C**) induced by LPS, and production of IL-4 (**D**), IL-6 (**E**) and IFN-γ (**F**) induced by PHA-L, was suppressed by addition of 1 µM cortisol. Increasing concentrations of RU-486 were added. Median values are indicated by the horizontal bars. Statistical significance is indicated at *p* < 0.05 (*), *p* < 0.01 (**) and *p* < 0.001 (***), relative to the negative controls (LPS or PHA-L, plus 1 µM cortisol), following repeated measures ANOVA and Dunnett’s multiple comparison test

RU-486 added at 3.3 μM was sufficient to reverse the inhibitory effect of 1 µM cortisol on LPS induction of IL-6 (Fig. 3A). However, 10 μM RU-486 was required to significantly reverse cortisol inhibition of LPS-induced IL-1β (Fig. 3B) or TNF-α production (Fig. 3C). Similarly, 10 µM RU-486 reversed the inhibition of IL-4 and IL-6 production by 1 µM cortisol in PHA-L-stimulated blood cultures (Fig. 3D and E), although both 1 µM and 3.3 µM RU-486 significantly reversed cortisol inhibition of IFN-γ production (Fig. 3F).

### RU-486 partially reverses innate but not T cell-induced cytokine responses in patients with SAH, with and without IL-1Ra therapy

**Fig. 4:**
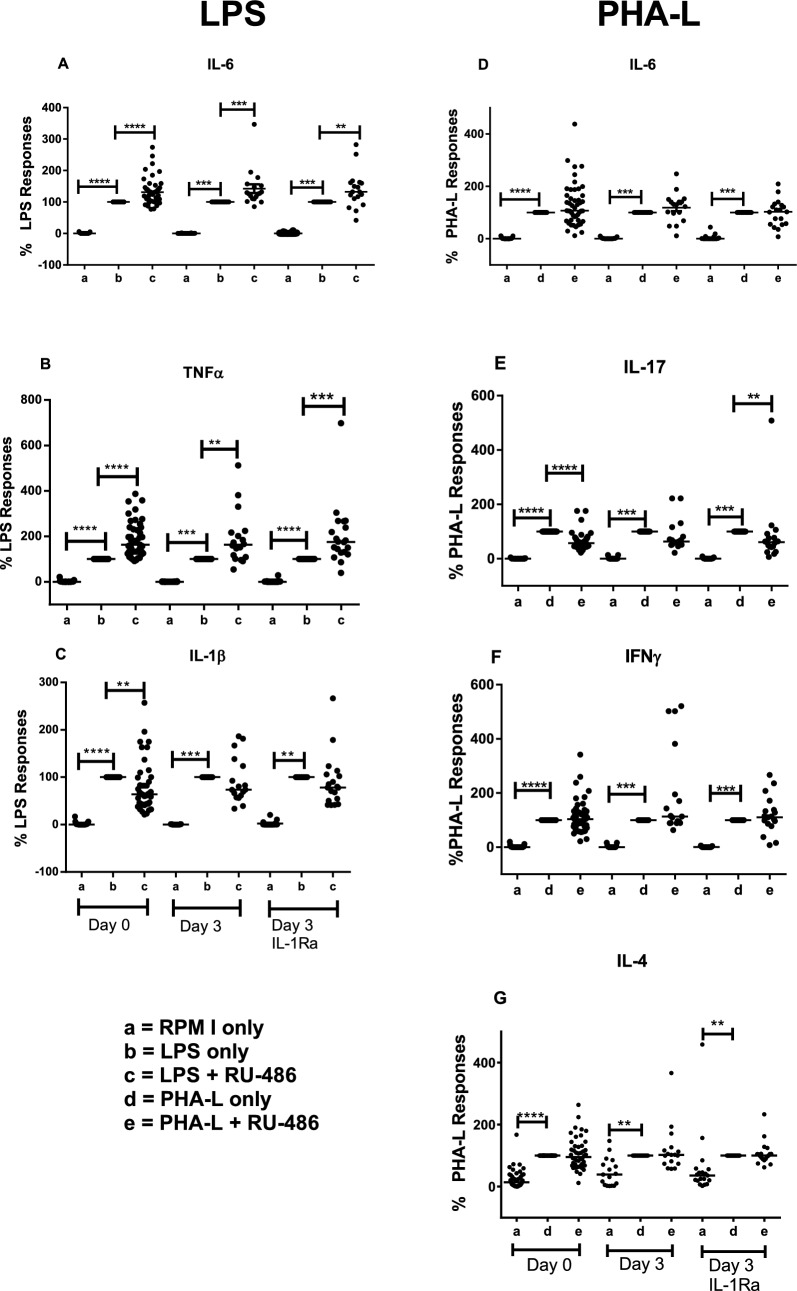
RU-486 increases innate, but not T lymphocyte cytokine responses in patients after SAH. Production of cytokines is expressed as % of induction by LPS (**A**–**C**) or PHA-L (**D**–**G**) alone. Statistical significance between groups is indicated at *p* < 0.01 (**), *p* < 0.001 (***) and *p* < 0.001 (****), Wilcoxon matched-pairs signed rank test was used

The effect of RU-486 was evaluated in blood cultures of 36 patients with SAH, 19 of whom were randomised to receive IL-1Ra after the first blood sample (day 0), and 17 controls who received no IL-1Ra. Addition of RU-486 to LPS-treated blood increased IL-6 and TNF-α production at day 0 (pre-IL-1Ra treatment), as well as in the control and IL-1Ra groups at day 3, when compared to LPS alone (Fig. 4A, B). In contrast, production of IL-1β decreased in the presence of RU-486, compared to LPS alone at day 0, and there was no significant effect at day 3 for either control or IL-1Ra groups (Fig. 4C). Addition of RU-486 to PHA-L-treated blood (Fig. 4D–G) had no significant effect on IL-6, IFN-γ or IL-4 production at day 0 or at day 3 in the control or IL-1Ra group, compared to PHA-L alone. RU-486 reduced IL-17 production at day 0 (*p* < 0.00001) and also at day 3 (*p* < 0.01) for the IL-1Ra group. Moderate, but significant inverse correlations were observed between plasma cortisol at baseline and IL-6 (*r* = − 0.40, *p* < 0.05), TNF-α (*r* = − 0.359, *p* < 0.05) and IL-1β (*r* = − 0.444, *p* < 0.01) in LPS-stimulated blood supernatants. In PHA-L-stimulated supernatants, only IL-6 showed a significant inverse correlation with cortisol (*r* = − 0.419, *p* < 0.05). The correlations for IL-17, IFN-γ and IL-4 were low (*r* ≤ − 0.25) and not statistically significant. There were no significant correlations between plasma cortisol and any cytokine concentrations at day 3 for the IL-1Ra or control groups.

**Fig. 5:**
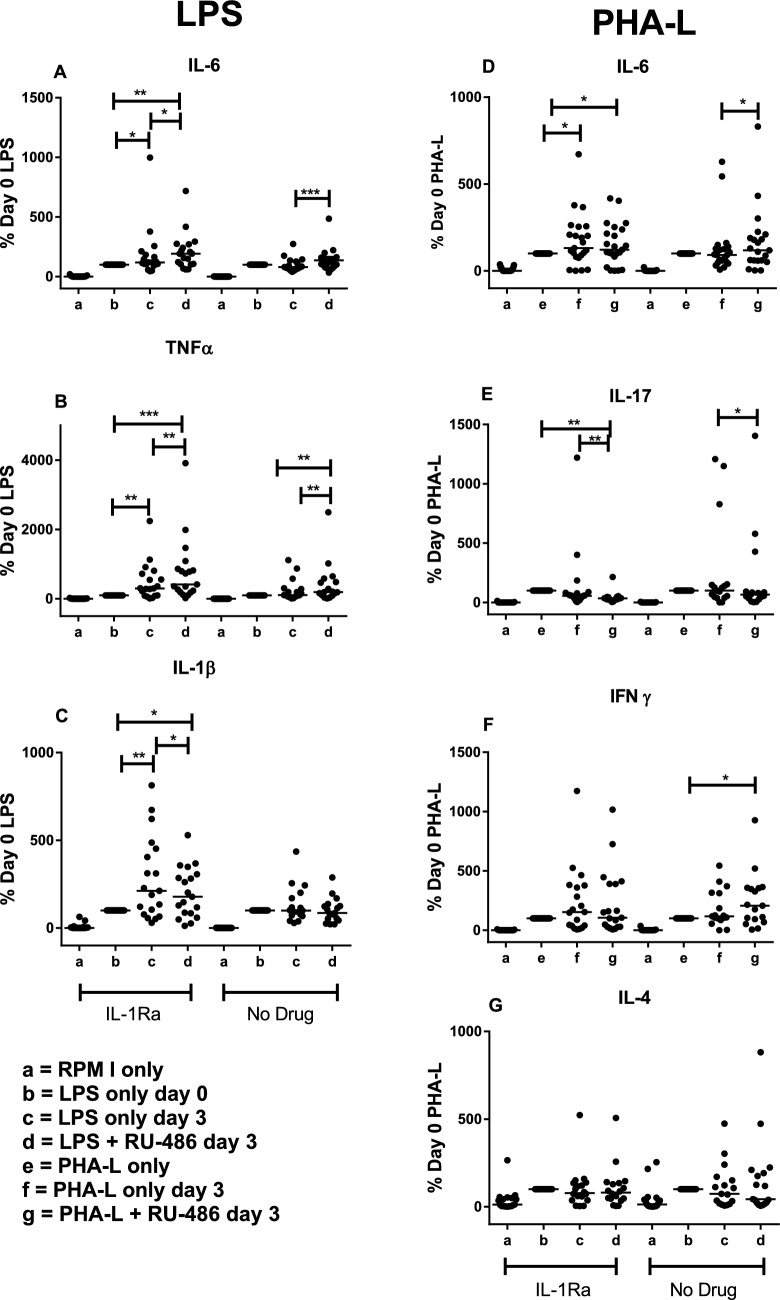
Differences between day 0 and day 3 LPS and PHA-L induced cytokine responses in IL-1Ra and control groups. Production of cytokines is expressed as % of induction by LPS (**A**–**C**) or PHA-L (**D**–**G**) alone at day 0. Statistical significance between groups is indicated at *p* < 0.05 (*), *p* < 0.01 (**) and *p* < 0.001 (***), Wilcoxon matched-pairs signed rank test was used

To evaluate the effect of cortisol inhibition after 3 days of IL-1Ra treatment, responses were examined relative to the LPS response at day 0. Comparison of the control and IL-1Ra treated groups at day 3, in the absence of RU-486, showed a difference only for IL-17, which was significantly reduced in the treated group….. (*p* < 0.05, not shown). Although the day 3 cytokine responses were not significantly different to the day 0 responses in the control group, LPS-induced cytokine responses were increased at day 3 in the IL-1Ra treatment group, compared to day 0 responses (Fig. 5). The additional impact of RU-486 was consistent with its effect on day 0 responses. In contrast, only the T lymphocyte IL-6 response was increased in the IL-1Ra group at day 3 relative to day 0 and this was not affected by RU-486. IL-17 responses were slightly lower at day 3 in the IL1-Ra group and RU-486 depressed these further, consistent with its effect at day 0. There were no significant differences between plasma cortisol concentrations at day 0 and day 3, or between the IL-1Ra group and the control group on day 3.

## Discussion

We have shown that suppression of innate immune cytokine responses occurs after SAH in a similar manner to that observed previously in ischaemic stroke [16] and this accords with a previous pilot study of patients with SAH [36]. The relationship between cytokine induction and cortisol was less clear for SAH patients than in those with ischaemic stroke. However, these two patient groups were evaluated differently. In the ischaemic stroke study, baseline samples were collected within 6 h of stroke, whereas baseline SAH samples were taken at any time up to 72 h following haemorrhage, due to differences in admission times. Therefore, any acute rises in cortisol that will have occurred during early trauma will have diminished in some SAH patients. Similarly, the effects of IL-1Ra were evaluated at 24 h and 5–7 days after the baseline stroke sample, but the day 3 sample in SAH could be between 1 and 3 days after IL-1Ra administration. Despite this variability, and the small numbers of patients in our initial evaluation, cortisol concentrations were greater than 0.33 µM in almost half the patients and only one control, and were also correlated with suppression of TNF-α. At 0.33 µM cortisol suppressed each of the evaluated cytokines, except IFN-γ. The variability in time of blood collection may have reduced the observed impact of cortisol, since it is known that injury disrupts normal diurnal rhythms and the observation that many patients had elevated cortisol supports the concept that it may have had an effect on their physiology [1, 54]

The study in healthy volunteers indicated that cortisol, at concentrations observed in SAH patient’s plasma, suppressed both innate and T lymphocyte-mediated arms of the peripheral immune response, with respect to cytokine production. The healthy volunteer study also showed that this suppression is reversed by blocking cortisol with the antagonist RU-486. The subsequent study of SAH patients recruited to a trial of IL-1Ra treatment supported the mediation of innate cytokine suppression by cortisol, since RU486 increased production of IL-6 and TNF in the presence of LPS. However, there was no evidence that T cell-mediated cytokine production was subject to cortisol regulation.

Glucocorticoids have long been implicated in suppression of inflammation and innate immunity, including the suppression of cytokine production by monocytes [4, 11, 28, 39, 45]. Glucocorticoids also suppress both the proliferation of and cytokine production in T-cell lymphocytes [5, 7, 42, 44]. Hence, cortisol can suppress both arms of the peripheral cellular immune response [17], although these responses have generally not been evaluated in terms of their relative susceptibility to cortisol and direct evidence for a pathophysiological role is limited.

We used a human whole blood culture system to reduce the impact of cell isolation procedures, and to maintain cells in a more physiological environment [10, 11, 25, 29]. Previous studies of the impact of cortisol on cellular immune responses have generally not been done in the context of a physiological medium, although DeRijk et al. [11] showed that cortisol reduced IL-1β and TNF-α, but not IL-6, production in whole blood cultures in relation to exercise stress.

There was considerable variation in cytokine production between individual healthy volunteers, which has been noted previously [10, 27, 43]. Variability in the sensitivity of different cytokines to cortisol was also apparent. Higher sensitivity of TNF-α to stress-induced levels of glucocorticoids has been noted previously, in comparison to IL-6, which was more resistant [11]. It was suggested that this was compatible with data indicating that IL-6 has an anti-inflammatory as well as a pro-inflammatory action [41]. Intragroup variation in cytokine sensitivity to cortisol among healthy participants may be attributable to factors such as gender [48], and lifestyle factors, such as smoking [47], which are known to influence glucocorticoid sensitivity. In our volunteers, this variation in sensitivity was not pronounced, since 0.3 µM cortisol suppressed the production of each cytokine induced by either LPS or PHA-L. However, to avoid the impact of this variability on analysis we evaluated cortisol and RU-486 effects relative to individual responses in the absence of drug.

Cortisol has been proposed to reduce the ability to fight infection after stroke [6, 9], traumatic brain injury [21, 26], spinal cord injury [32, 40] and other pathologies [52]. An association of hypercortisolism with poor outcome has been noted following stroke and other brain injuries [30, 33]. This, and the potential link to infection susceptibility, suggests that reducing the immunosuppressive effect of cortisol may have therapeutic value, if appropriately targeted. However, there has been little direct evidence that blocking the impact of cortisol increases responsiveness of immune cells in patients exhibiting immune suppression. GR antagonists, and more recent selective compounds, have been proposed to treat infection [3, 34, 35]. RU-486 has been shown previously to reverse the suppressive effects of cortisol on the T cell-mediated response of isolated blood cells in serum-free medium, although in those experiments it was itself inhibitory at 10 µM [44]. This inhibition was not seen in our study, which used a lower concentration of ethanol (1% rather than 2.5%) and used whole blood instead of isolated peripheral blood mononuclear cells grown in serum-free medium. We found that RU-486 at 1 and 10 µM fully reversed the suppression of cytokine production caused by 1 µM cortisol in both LPS- and PHA-L-stimulated whole blood, suggesting that the use of such antagonists could well boost immune capacity that has been reduced by cortisol.

RU-486 increased IL-6 and TNF-α production induced by LPS in cells from SAH patients and this was also apparent after patients had been treated with IL-1Ra. However, unlike the blood responses of healthy volunteers in the presence of cortisol, RU-486 did not increase IL-1β production in cells from SAH patients. In this respect it is interesting that the tightest correlation between cortisol and a cytokine was with TNF. T lymphocyte-mediated responses of cells from patients were also unaffected by RU-486 in contrast to responses of blood cells from healthy volunteers, where RU-486 reversed cortisol induced suppression of IL-4, IL-6 and IFN-γ production that had been suppressed by cortisol. There could be several reasons for this. In healthy volunteers, RU-486 and cortisol were added at the same time, whereas in the SAH patients there was a delay of up to 72 h between haemorrhage and the addition of RU-486 to the blood. This variable delay could contribute to the variability of the data. The varied aetiology and pathophysiology of SAH in patients presents further complexity compared to the physiology of healthy volunteers and there may be other factors involved: e.g. some cytokines such as IL-6 and CRP already being raised in the plasma of patients [19].

Unlike our study with stroke patients [16], no significant difference was seen between the IL-1Ra and control groups for production of any of the cytokines stimulated by LPS or PHA-L. This may be attributable to the difference in timing of IL-1Ra administration, which was considerably delayed in SAH patients, relative to the ischaemic stroke trial of IL-1Ra. However, IL-6, TNF-α and IL-1β production in LPS-stimulated blood, and IL-6 production in PHA-L-stimulated blood was greater at day 3 than baseline, in the SAH patient group treated with IL-1Ra, whilst no such increase was observed for the control SAH patient group.

Although there are reasons to suppose that corticosteroids might be important in terms of reducing inflammation or maintaining other physiological responses, recent trials have failed to demonstrate any therapeutic effect of administered corticosteroid and suggested they may in fact result in a poorer outcome [13].

## Conclusion

These studies confirm that cytokine production by innate immune cells, following SAH, is reduced relative to that in healthy controls and the reduction correlates with increases in plasma cortisol. Cortisol, at concentrations similar to that found in plasma of SAH patients, suppresses the induction of cytokines by innate and adaptive immune cells in human blood from healthy volunteers and was effectively reversed by the GR antagonist, RU-486. However, in SAH patients, where cortisol was pathophysiologically induced, RU-486 reversed the suppression of TNF-α and IL-6 production by innate immune cells, including in patients treated with IL-1Ra. Further studies may be warranted where samples are collected more proximal to haemorrhage, to confirm and extend these observations, since the potential use of therapeutic measures to maintain an early and effective innate immune response in peripheral tissues could contribute to reducing post traumatic infections.

## Data Availability

All data generated or analysed during these studies are included in the published article.

## Abbreviations

SAH: Subarachnoid haemorrhage
PHA: Phytohaemagglutinin
IL: Interleukin
TNFα: Tumour necrosis factor alpha
INFƔ: Interferon gamma
ANOVA: Analysis of variance
ELISA: Enzyme-linked immunosorbent assay
